# Online Monitoring Oxidative Products and Metabolites of Nicotine by Free Radicals Generation with Fenton Reaction in Tandem Mass Spectrometry

**DOI:** 10.1155/2013/189162

**Published:** 2013-07-25

**Authors:** Shih-Shin Liang, Yow-Ling Shiue, Chao-Jen Kuo, Su-Er Guo, Wei-Ting Liao, Eing-Mei Tsai

**Affiliations:** ^1^Center for Resources, Research and Development, Kaohsiung Medical University, 100 Shih-Chuan 1st Road, Kaohsiung 80708, Taiwan; ^2^Department of Biotechnology, College of Life Science, Kaohsiung Medical University, 100 Shih-Chuan 1st Road, Kaohsiung 80708, Taiwan; ^3^Institute of Biomedical Science, National Sun Yat-Sen University, 70 Lienhai Road, Kaohsiung 80424, Taiwan; ^4^Graduate Institute of Medicine, Kaohsiung Medical University, 100 Shih-Chuan 1st Road, Kaohsiung 80708, Taiwan; ^5^Department of Nursing, Chang Gung University of Science and Technology, 2 Chia-Pu Road, Chiayi 61363, Taiwan

## Abstract

In general, over 70% absorbed nicotine is metabolized to cotinine and trans-3′-hydroxycotinine by cytochrome oxidase P450, and nicotine is also a major addictive and the psychoactive component in cigarettes. As a xenobiotic metabolism, hydrophobic compounds are usually converted into more hydrophilic products through enzyme systems such as cytochrome oxidase P450, sulfotransferases, and UDP-glucuronosyltransferases to deliver drug metabolites out of the cell during the drug metabolic process. In this study, an electrodeless electrochemical oxidation (EEO) reaction via Fenton reaction by producing free radical to react with nicotine to immediately monitor the oxidative products and metabolic derivatives of nicotine by tandem mass spectrometer (MS) is done. Fenton reaction generates free radicals via ferrous ion (Fe^2+^) and hydrogen peroxide (H_2_O_2_) to oxidize DNA and to degrade proteins in cells. In the EEO method, the oxidative products of nicotine including cotinine, cotinine-*N*-oxide, *trans*-3′-hydroxycotinine, nornicotine, norcotinine, 4-oxo-4-(3-pyridyl)-butanoic acid, 4-hydroxy-4-(3-pyridyl)-butanoic acid, and nicotine-*N′*-oxide were detected by tandem mass spectrometer to simulate the changes of nicotine and its derivatives in a time-dependent manner.

## 1. Introduction

 A free radical can be defined as an atom, molecule, or ion with an unpaired valence electron [1] and has a strong reactivity to attack other molecules or generate new free radicals by atom transfer radical polymerization (ATRP) [2]. Free radicals, such as superoxide, nitric oxide (NO), thyl, peroxyl, and hydroxyl radical, play important roles in biological processes [3], and these oxygen-containing free radicals usually originate from losing a partial valence electron in electron transport chain at mitochondria. The terms “reactive oxygen species (ROS)” and “reactive nitrogen species (RNS)” contain not only free radicals but also active reagents such as hydrogen peroxide (H_2_O_2_), singlet oxygen, and ozone (O_3_) in living organisms.

ROS- and RNS-related oxidative stress resulted in disorders including serious aging, cancers, stroke, and diabetes [4, 5]. According to the previous reports, free radicals were also involved in neurodegenerative disease such as Alzheimer's disease and Parkinson's disease [6]. However, not all of the free radicals play harmful roles in human health. Nitric oxide (NO), generated by nitric oxide synthases (NOSs) when L-arginine is converted to citrulline, serves as a cellular signaling molecule to regulate vasodilatation in blood vessels by activating guanylate cyclase, guanosine 3′,5′-monophosphate (cyclic GMP), and protein kinase G to relax smooth muscle via proteins phosphorylation [7].

In the last decade, metabolomics has progressed at a marvelous rate in the omics field. At an early stage of drug development, rat liver microsomes (RLMs) with specific cytochrome P450 (CYP450) activity were employed as an approach for the investigation of drug metabolism [8, 9]. However, in drug discovery, it is time consuming, labor consuming, and expensive for the target molecules screening, pharmacodynamics, and pharmacokinetics followed by a series of *in vivo* and *in vitro* experiments. Fortunately, Volk et al. utilized on line electrochemical cell coupled with tandem mass spectrometer (EC-MS) to proceed with new instrumental analyses for oxidative metabolic molecules [10–13]. Furthermore, Karst's group had extended the application of EC-MS to connect with liquid chromatographic (LC) system by a switch valve to be an EC-LC-MS [14]. The EC-LC-MS system is not described as a continuous analysis but as an online sequential separation [14, 15]. Moreover, Karst's group had utilized EC-MS techniques to simulate the metabolic pathways of drug such as a muscle relaxant tetrazepam and an antiarrhythmic drug procainamide [14, 16], to investigate and identify nucleotide oxidative products [17], and to analyze the oxidation of aniline with the formation of protein adducts [18]. Moreover, the cyclic voltammetry (CV) technique has been utilized to evaluate redox reaction of various biomolecules with their oxidative derivatives and to compare the oxidative products by different electrodes [19–25].

**Figure 1:**
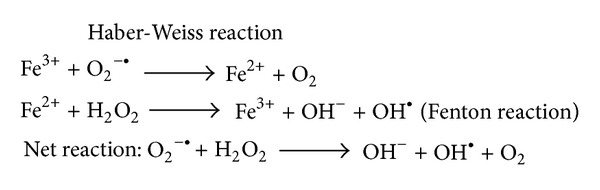
The Haber-Weiss Reaction and Fenton reaction for hydroxyl free radical generation.

In this study, we generated free radicals by Fenton reaction (Figure 1) to investigate the oxidative products or possible metabolites of nicotine with an online sequential analytic mass spectrometer system. Besides, Fenton reaction is an oxidative reaction occurring in mitochondria when ferrous ion (Fe^2+^) reacts with H_2_O_2_ to generate free radicals such as the hydroxyl radical (OH^·^) and superoxide anion. Meanwhile, superoxide dismutase (SOD) converts superoxide anion to H_2_O_2_. The total reaction is described as Haber-Weiss reaction (Figure 1), and the net reaction shows that superoxide anion and H_2_O_2_ are converted to oxygen, hydroxide ion, and hydroxyl radical. Avoiding contamination of electrodes and crack of electrochemical flow cell (max. pressure: 40 psi, Antec Leyden, Zoeterwoude, The Netherlands) [14, 15, 17, 18] caused by high pressure of HPLC, online electrodeless electrochemical oxidation (EEO) of free radicals generated by Fenton reaction was a novel technique to monitor the oxidative products and metabolites of nicotine. The EEO/HPLC separated system was equipped with electrospray ionization (ESI) and tandem mass spectrometer (MS/MS) to be an online EEO/HPLC ESI-MS/MS monitoring system. According to this EEO/HPLC ESI-MS/MS electrochemical equipment, the metabolic derivatives of nicotine [26] including cotinine, cotinine-*N*-oxide, *trans*-3′-hydroxycotinine, nornicotine, norcotinine, nicotine-*N*′-oxide, 4-oxo-4-(3-pyridyl)-butanoic acid, and 4-hydroxy-4-(3-pyridyl)-butanoic acid were detected by tandem mass spectrometer. 

## 2. Materials and Methods

### 2.1. Chemicals

The reagents including hydrogen peroxide (H_2_O_2_), nicotine, sodium acetate (CH_3_COONa), and potassium titanium oxide oxalate dehydrate (C_4_O_9_K_2_Ti·2H_2_O) were provided by Sigma-Aldrich (St. Louis, MO, USA). The concentrated sulfuric acid, anhydrous methanol (MeOH), and ferrous sulfate heptahydrate (FeSO_4_·7H_2_O) were purchased from J. T. Baker (Phillipsburg, NJ, USA). The chemicals such as 1, 10-phenanthroline monohydrate (C_12_H_8_N_2_·H_2_O), acetic acid, and ammonium acetate (NH_4_CH_3_COO) were purchased from Aldrich (Milwaukee, WI, USA). Distilled water was prepared to 18.2 MΩ cm resistivity at 25°C by a Milli-Q system (Millipore, Bedford, MA).

### 2.2. The Concentration of H_2_O_2_ and Fe^2+^ Solutions

 The concentration of H_2_O_2_ was determined by Sellers' method [27, 28]. The reagent of potassium titanium (IV) oxalate (K_2_TiO(C_2_O_4_)·2H_2_O 354 mg was mixed with 2.72 mL of concentrated sulfuric acid and 3 mL of deionized water. After the diluted sulfuric acid solution was cooled to room temperature, it was adjusted to 10 mL by distilled water. Titanium(IV) solution 500 *μ*L was composed of different concentration of H_2_O_2_ solutions (500 *μ*L) and displayed yellow-orange complex detected by spectrophotometer at 400 nm. The calibration curve was plotted by the intensities of spectrophotometer of different H_2_O_2_ concentration.

The concentration of ferrous ion was determined by ferroin indicator. Ferroin indicator includes both solution A (100 mg of 1,10-phenanthroline monohydrate dissolved in 100 mL distilled water) and solution B (25 g of ammonium acetate mixing with 15 mL distilled water and 70 mL acetic acid). The different concentrations of ferrous solutions (1 mL) were prepared with 40 *μ*L solution A and 100 *μ*L solution B, respectively. After 25 mins, solutions were detected by spectrophotometer at 510 nm. The calibration curve was plotted by the intensities of spectrophotometer at different ferrous sulfate concentrations.

### 2.3. The Preparation of Fenton Reaction and Nicotine

 The reactive solution composed of 10 *μ*L nicotine (20 ppm), 90 *μ*L H_2_O_2_ (3%), 1890 *μ*L sodium acetate buffer (50 mM, pH 5.6), and 10 *μ*L ferrous sulfate heptahydrate (100 mM) solutions was adjusted to 200 ppb nicotine, 0.135% H_2_O_2_, and 0.5 mM FeSO_4_. The ultrahigh performance liquid chromatography (UHPLC) gradient was set at 30 mins in one experiment. The sequential analyses were performed by injection of 10 *μ*L nicotinic oxidative mixture via the syringe of autosampler every 30 mins. 

### 2.4. UHPLC and ESI-MS Analytic Conditions

 Immediate online electrospray ionization mass spectrometry (ESI-MS) analyses of mixture with nicotine and Fenton reaction reagents were detected by a Thermo Finnigan TSQ Quantum Ultra Mass Spectrometer Analytic System (Thermo Fisher Scientific Inc., Waltham, MA, USA) equipped with the Micro ESI ion source which was set at 3.0 kV coupled with Acella 1250 UHPLC system (Thermo Fisher Scientific Inc., Waltham, MA, USA). The oxidative mixture was subject directly into the UHPLC via Acella 1250 autosampler and was separated by Shiseido HPLC CAPCELL PAK C18 MGII column (150 mm × 1.5 mm, 3.0 *μ*m, Tokyo, Japan). The UHPLC flow rate was set at 250 *μ*L/min (gradient pump). The mobile phases were composed of (A) 10 mM NH_4_CH_3_COO in water and (B) 10 mM NH_4_CH_3_COO in 100% MeOH with a linear gradient followed from 5% (B) in 2 min, 5%–40% (B) in 20 min, 40%–98% in 5 min, 98% (B) in 2 min, 98%–5% (B) in 0.1 min, and 5% (B) in 2.9 min. The nicotine, oxidative chemicals, and its metabolic derivatives were detected by mass spectrometer with applying voltage of 3.0 kV in the positive ion mode, vaporizing and capillary temperature set at 300°C and 350°C, respectively, sheath gas and aux gas pressure set at 35 and 10, respectively, collision pressure at 1.5, and collision energy adjusted at 25 V. The survey scan mode was set at *m/z* 50–250 Da in the Quadrupole I chamber and nicotinic oxidative ions were detected and selected (intensity > 10^4^) in MS mode with three high intensity signals (data dependant scan), transferred into collision-induced dissociation (CID) chamber for MS/MS fragmentation and further detected in Quadrupole III chamber. The Xcalibur software (version 2.2, Thermo-Finnigan Inc., San Jose, CA) was utilized to control and adjust mass spectrometry instrument and data acquisition.

## 3. Results and Discussion

 In this study, Fenton reaction generated free radical to react with nicotine and produced nicotinic oxidative products and derivatives. The electrodeless electrochemical oxidation (EEO) technique integrated with tandem mass spectrometer (EEO/UHPLC-ESI-MS/MS) for oxidative derivatives monitoring. By MS characterization, the results of nicotine and its derivatives were listed in Table 1.

### 3.1. Free Radical Generation from Haber-Weiss Reaction

 As a catalytic reagent, Fe^2+^ reacted with H_2_O_2_ for producing hydroxyl free radical and oxygen. In the process of free radical generation, bubbles (O_2_) were produced from the sample bottle. The Fenton reaction generating hydroxyl free radical was a side reaction of Haber-Weiss reaction. The chemical reaction formulas of Haber-Weiss reaction and Fenton reaction were listed in Figure 1.

### 3.2. EEO/UHPLC ESI-MS/MS Equipment for Nicotine and Its Oxidative Derivatives Monitoring

**Figure 2:**
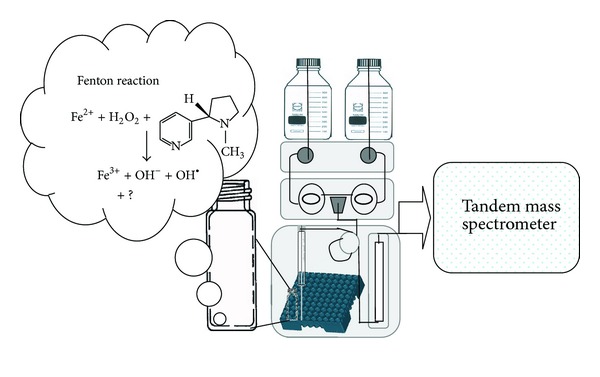
The schematic representations of experimental apparatus and Fenton reaction oxidative processes. Nicotine reacted with hydroxyl free radical in sample bottle and nicotinic oxidative products or metabolic derivatives were injected via syringe into analytic column in autosampler.

**Figure 3:**
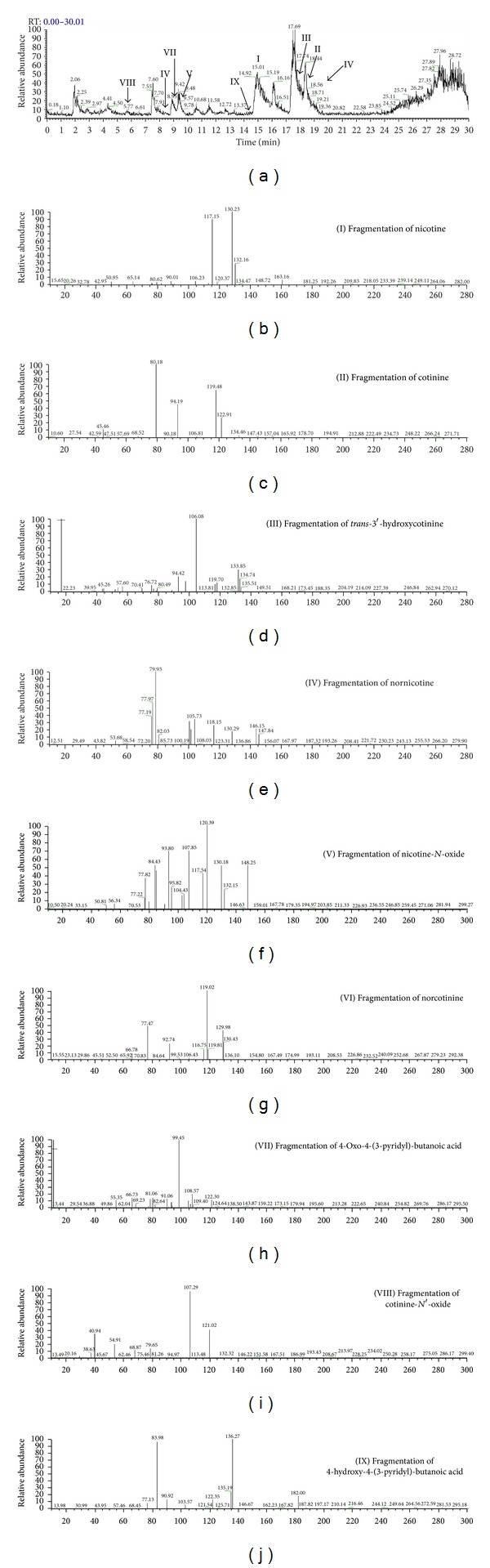
Electrodeless electrochemical oxidation (EEO) method coupled with UHPLC-MS/MS showed the spectra of the nicotine and its derivatives labeled with I–IX. The spectra displayed (a) base peak chromatogram and fragmentation pattern of nicotine (b), cotinine (c), *trans*-3′-hydroxyl cotinine (d), nornicotine (e), nicotin-*N*-oxide (f), norcotinine (g), 4-oxo-4-(3-pyridyl)-butanoic acid (h), 4-hydroxy-4-(3-pyridyl)-butanoic acid (i), cotinine-*N*′-oxide (j).

**Figure 4:**
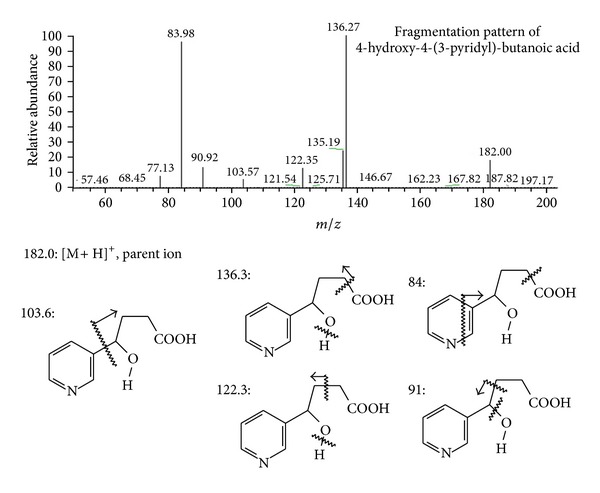
ESI-MS/MS fragmentation pattern spectrum of nicotinic derivative: 4-hydroxy-4-(3-pyridyl)-butanoic acid.

 The schematic representation of experimental apparatus was showed in Figure 2. The mixture of free radical and nicotinic derivatives was injected by the syringe of autosampler into UHPLC system. In this technique, nicotine and its oxidative derivatives could be monitored in a run-to-run and time-dependent manner (data not shown). According to the previous studies [29–31], nicotinic metabolites detected in hair, urine, and plasma, and nicotine, 3-hydroxycotinine, or cotinine were selected as candidates. However, in the EEO/UHPLC ESI-MS/MS method, nicotinic metabolites including cotinine, cotinine-*N*-oxide, *trans*-3′-hydroxycotinine, nornicotine, norcotinine, nicotine *N*′-oxide, 4-oxo-4-(3-pyridyl)-butanoic acid, and 4-hydroxy-4-(3-pyridyl)-butanoic acid were identified. The characterization of nicotine and its derivatives with name of derivatives, molecular formula, molecular weight, *m/z* of parent ion, and daughter ions was listed in Table 1. Furthermore, UHPLC base peak chromatogram and the fragmentation pattern of each metabolite were shown in Figure 3. The fragmentation patterns of nicotine and its derivatives showed that each derivative was selected by mass spectrometer (depending on different molecular weights) and fragmentized by collision gas with kinetic energy in collision chamber. The fragments (daughter ions) of MS/MS data labeled with English vocabulary from (b) to (j) were shown in Figure 3 and Table 1. We selected nicotinic metabolic reagent, 4-hydroxy-4-(3-pyridyl)-butanoic acid, as a representative; the fragmentation pattern and predictive fragments of this metabolite were shown in Figure 4. According to Figure 3, in the fragmentation patterns of nicotine, cotinine, *trans*-3′-hydroxycotinine, nornicotine, nicotine *N*′-oxide, and cotinine-*N*′-oxide, the spectra included the pyridinium ion at *m/z* 80. Unfortunately, the pyridinium ion could not be observed at the fragmentation patterns of 4-hydroxy-4-(3-pyridyl)-butanoic acid and 4-oxo-4-(3-pyridyl)-butanoic acid. However, in the predictive fragments of 4-hydroxy-4-(3-pyridyl)-butanoic acid spectra, we could track the sources of each daughter ion. To sum up, EEO/UHPLC ESI-MS/MS method could be utilized to simulate the generation of metabolites and to monitor the changes in a time-dependent manner.

### 3.3. Establishment of Nicotinic Oxidative Pathway

**Figure 5:**
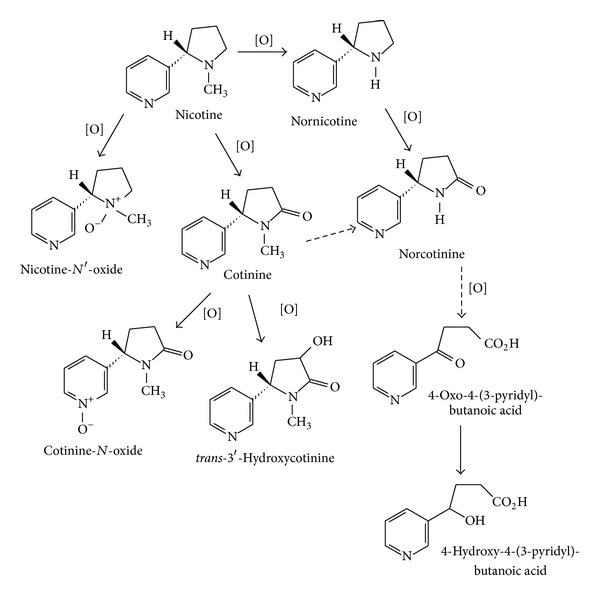
Pathways establishment of nicotine metabolism in electrodeless electrochemical oxidation (EEO) method coupled with UHPLC ESI-MS/MS.

 According to the previous study [26] and the spectra shown in Figures 3 and 4 and Table 1, we could establish the oxidative pathway of nicotine. Moreover, the nicotinic oxidative metabolites characterized by EEO/UHPLC ESI-MS/MS could be integrated into the metabolism pathway, further shown in Figure 5. The arrow with a solid line directly showed the metabolic processes and the arrow with a dotted line indicated that this pathway required an intermediate. The label of [O] showed that the metabolite was generated via oxidative reaction.

## 4. Conclusion

 The establishment of nicotinic metabolic pathway based on the electrodeless electrochemical oxidation (EEO) utilizing Fenton reaction to produce hydroxyl free radical to react with nicotine was demonstrated. The online EEO/UHPLC ESI-MS/MS analytic system setup could be employed in metabolomics study. Thus, nicotinic metabolic pathway was established via the characterized oxidative nicotinic derivatives. The tandem mass spectrometer is a powerful analytic instrument with characterization of nicotinic derivatives by fragmentation patterns. However, by the sequential analyses, this analytic technique could not immediately monitor the changes of nicotinic metabolites. In the future, Fenton reaction (Fe^2+^/H_2_O_2_) has the potential to be utilized in exploring oxidative products of biomolecules including DNA, small molecular drugs, and formation of protein adducts.

## Figures and Tables

**Table 1:** Metabolic derivatives of nicotine in EEO/ESI-MS/MS. The molecular formula, molecular weight, and *m*/*z* of parent ion and daughter ions are listed.

Name	Molecular formula	Molecular weight (Da)	Parent ion	Daughter ions
Nicotine	C_10_H_14_N_2_	162.12	162.2	117, 130, 132
Cotinine	C_10_H_12_N_2_O	176.22	177.7	80, 119.5, 134.5
*trans*-3′-Hydroxyl cotinine	C_10_H_12_N_2_O_2_	192.21	194.0	106, 118, 134
Nornicotine	C_9_H_12_N_2_	148.20	148.7	80, 130
Nicotine-*N*-oxide	C_10_H_14_N_2_O	178.23	179.0	120, 130, 148
Norcotinine	C_9_H_10_N_2_O	162.19	162.5	67, 93, 119
4-Oxo-4-(3-pyridyl)-butanoic acid	C_9_H_9_NO_3_	179.17	180.2	81, 99.5, 108.6
4-Hydroxy-4-(3-pyridyl)-butanoic acid	C_9_H_11_NO_3_	181.07	182.2	84, 91, 122, 136
Cotinine-*N*′-oxide	C_10_H_12_N_2_O_2_	192.21	192.9	80, 121
